# Toward drift-free high-throughput nanoscopy through adaptive intersection maximization

**DOI:** 10.1126/sciadv.adm7765

**Published:** 2024-05-23

**Authors:** Hongqiang Ma, Maomao Chen, Phuong Nguyen, Yang Liu

**Affiliations:** ^1^ Department of Medicine, Department of Bioengineering, University of Pittsburgh, Pittsburgh, PA 15213, USA.; ^2^ Department of Bioengineering, Beckman Institute for Advanced Science and Technology, University of Illinois Urbana-Champaign, Urbana, IL 61801, USA.; ^3^ Department of Bioengineering, Department of Electrical and Computer Engineering, Beckman Institute for Advanced Science and Technology, Cancer Center at Illinois, University of Illinois Urbana-Champaign, Urbana, IL 61801, USA.

## Abstract

Single-molecule localization microscopy (SMLM) often suffers from suboptimal resolution due to imperfect drift correction. Existing marker-free drift correction algorithms often struggle to reliably track high-frequency drift and lack the computational efficiency to manage large, high-throughput localization datasets. We present an adaptive intersection maximization-based method (AIM) that leverages the entire dataset’s information content to minimize drift correction errors, particularly addressing high-frequency drift, thereby enhancing the resolution of existing SMLM systems. We demonstrate that AIM can robustly and efficiently achieve an angstrom-level tracking precision for high-throughput SMLM datasets under various imaging conditions, resulting in an optimal resolution in simulated and biological experimental datasets. We offer AIM as one simple, model-free software for instant resolution enhancement with standard CPU devices.

## Teaser

Adaptive intersection maximization-based drift correction instantly enhances image resolution in high-throughput nanoscopy.

## INTRODUCTION

Pushing the resolution limit is one of the most important pursuits of single-molecule localization microscopy (SMLM). Over the past decade, major efforts have been expended to achieve its optimal resolution through various technical innovations, such as maximizing photon budget (*1*, *2*), integrating patterned illumination (*3*, *4*), optimizing noise model (*5*, *6*), and point spread function model (*7*). Sample drift is a long-standing problem in SMLM that can directly degrade the resolution of the reconstructed super-resolution image. Marker-free drift estimation approaches such as redundant cross-correlation (RCC) (*8*) are routinely performed in SMLM due to their simplicity without the need for system modification and special sample preparation (*9*). The recently reported methods such as drift at minimum entropy (DME) (*10*) and mean shift algorithm (*11*) continue improving the tracking accuracy for high-frequency drift. Despite these advancements, current drift correction methods lack sufficient precision, speed, or robustness to compensate for high-frequency drift, which is one of the main factors preventing SMLM from reaching their theoretically achievable resolution (*2*, *12*). This challenge is further exacerbated when dealing with the large datasets typical of high-throughput SMLM systems, which are becoming increasingly common (*13*, *14*). In attempts to mitigate these issues, compromised solutions such as reduced tracking frequency or selection of a small region of interest (ROI) from the large dataset are used, both of which can lead to substantial information loss. In addition, the performance of some algorithms heavily relies on the optimal parameter settings (*10*) that may vary for different datasets.

To achieve ultrahigh (e.g., sub-10 nm) image resolution or for ultraprecise distance measurement, marker-assisted drift correction (*2*, *12*) is essential. This method works well in total internal reflection fluorescence (TIRF) microscopy when fiducial markers and imaging targets share the same focal plane, as long as the markers present minimal interference with the imaging targets and remain stable throughout the entire image acquisition process. However, challenges arise when markers and targets are on different focal planes, necessitating additional dedicated detection optics or workflow for robust marker position tracking, thereby increasing the complexity of the instrument (*2*, *12*, *15*). Therefore, it remains a major challenge for marker-free drift correction approaches to achieve drift-free image reconstruction for the optimal resolution comparable to the state-of-the-art marker-assisted drift correction in SMLM.

Here, we present a marker-free drift correction method, referred to as adaptive intersection maximization (AIM) for high-throughput SMLM. AIM is model free and easy to use, it can robustly achieve high precision drift correction with a high tracking frequency with minimal parameter tuning. Through numerical simulation and biological experiments, we demonstrate that AIM can achieve a sub-angstrom (<0.1 nm) drift tracking error under various scenarios. Our results also indicate the importance of correcting high-frequency drift in achieving the optimal spatial resolution for SMLM. AIM can improve the spatial resolution by a factor of two by precisely correcting both low-frequency and high-frequency drift, which is important in ultraprecise structural measurement.

## RESULTS

### General principle of AIM

**Fig. 1.**
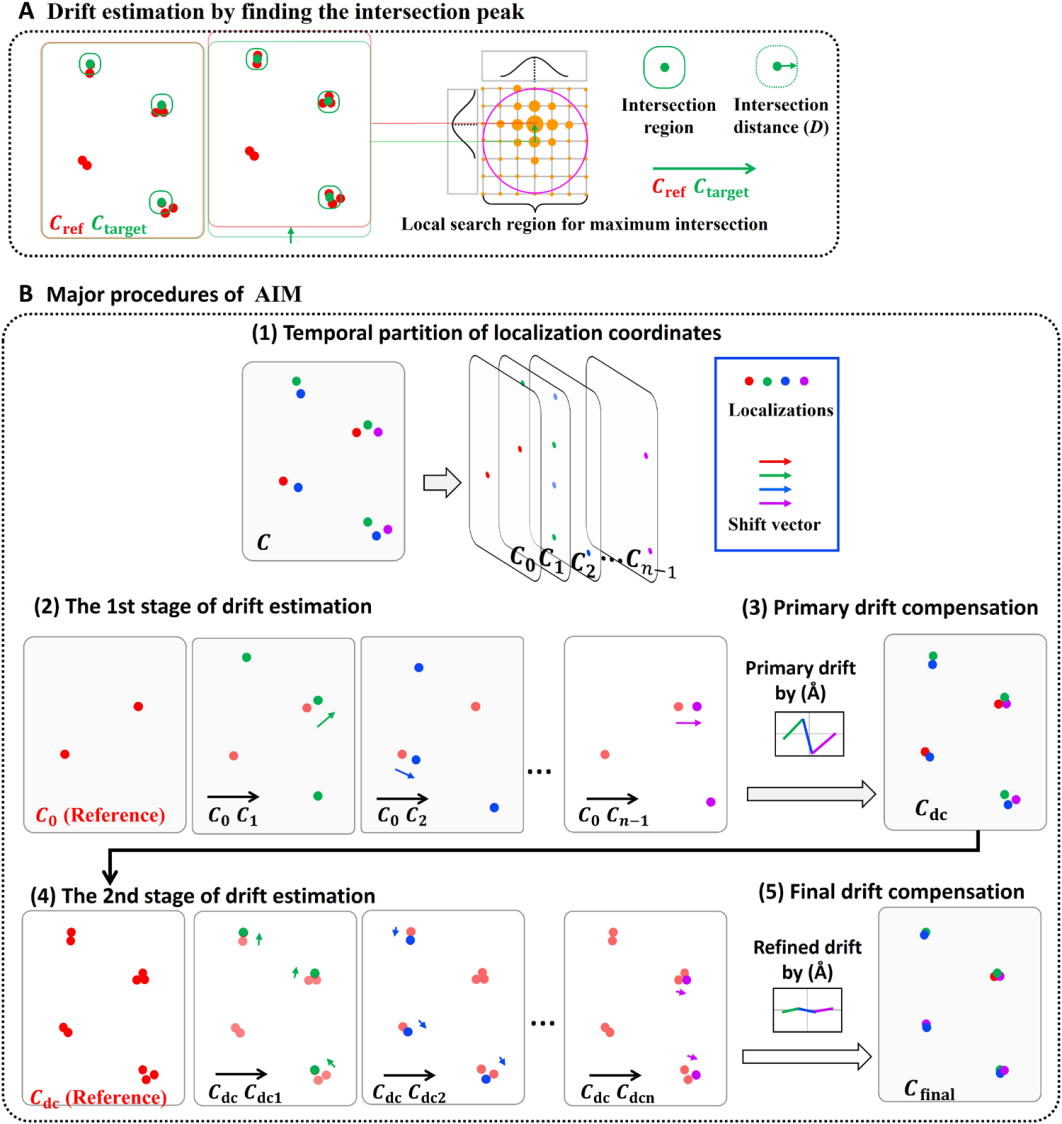
Schematic illustrating the principle of AIM-based drift correction method. (**A**) Our drift estimation approach is based on finding the position of the intersection map with the maximum value. (**B**) The main procedures of AIM use a two-stage adaptive processing strategy. (1) The localizations are temporally segmented into *n* parts (*C*_0,_ *C*_1_, *C*_2_…*C*_*n*−1_). (2 and 3) The initial stage generates a preliminary drift estimated by maximizing the intersection of localizations between the reference (e.g., *C*_0_) segment and other temporal segments (*C*_1_, *C*_2_…*C*_*n*−1_). (4 and 5) The entire dataset (*C*_dc_), now preliminarily corrected for drift, is then used as a new reference for a second, more refined stage of drift estimation. This process retains the full information content of the entire dataset and enhances the precision and robustness.

In SMLM, one super-resolution image is composed of a set of localization coordinates for fluorescent emitters acquired during a long period of image acquisition, spanning tens of thousands of frames to accumulate a sufficient number of localizations. In such a dataset, it is common for multiple localizations to originate from the same emitters, recorded across various temporal instances. Ideally, in a drift-free system, the coordinates of these localizations originating from the same emitters can be repeatedly detected in multiple image frames acquired at different time points and should be “intersected” within a threshold distance determined by localization precision, as illustrated in Fig. 1. The spatial drift reduces the number of these intersected localizations obtained at different time points, thus resulting in a lower number of intersected localizations.

AIM introduces a drift correction approach by maximizing the intersection of localization pairs across temporally distinct datasets (Fig. 1A), enabling accurate estimation of spatial drift. Conventional methods partition the entire dataset into many (*n*) temporally segmented blocks (Fig. 1B), using the partial subset as the reference for registration across blocks via image correlation (*8*) or entropy minimization (*10*). While effective, these methods are limited by their dependence on the partial data (1/*n* of the entire dataset), which can compromise their robustness and precision, especially for high-frequency drift where the number of localizations at each temporal segment is low. AIM addresses these limitations via a two-stage adaptive processing strategy. The initial stage generates a preliminary drift estimated by maximizing the intersection of localizations across temporal segments (Fig. 1A). Following this, the entire dataset, now preliminarily corrected for drift, is used as a new reference for a second, more refined stage of drift estimation (Fig. 1B). This process retains the full information content of the entire dataset and enhances the precision and robustness.

Moreover, distinct from complex image-based correlation or other optimization methods, AIM, centered around the counting of intersected localization points, stands out for its computational efficiency and robustness. Figure S1 further illustrates that the precision of drift tracking is enhanced with the increase in the image size and the number of intersected localizations, attributing to the greater information content leveraged.

### Numerical simulations

**Fig. 2.**
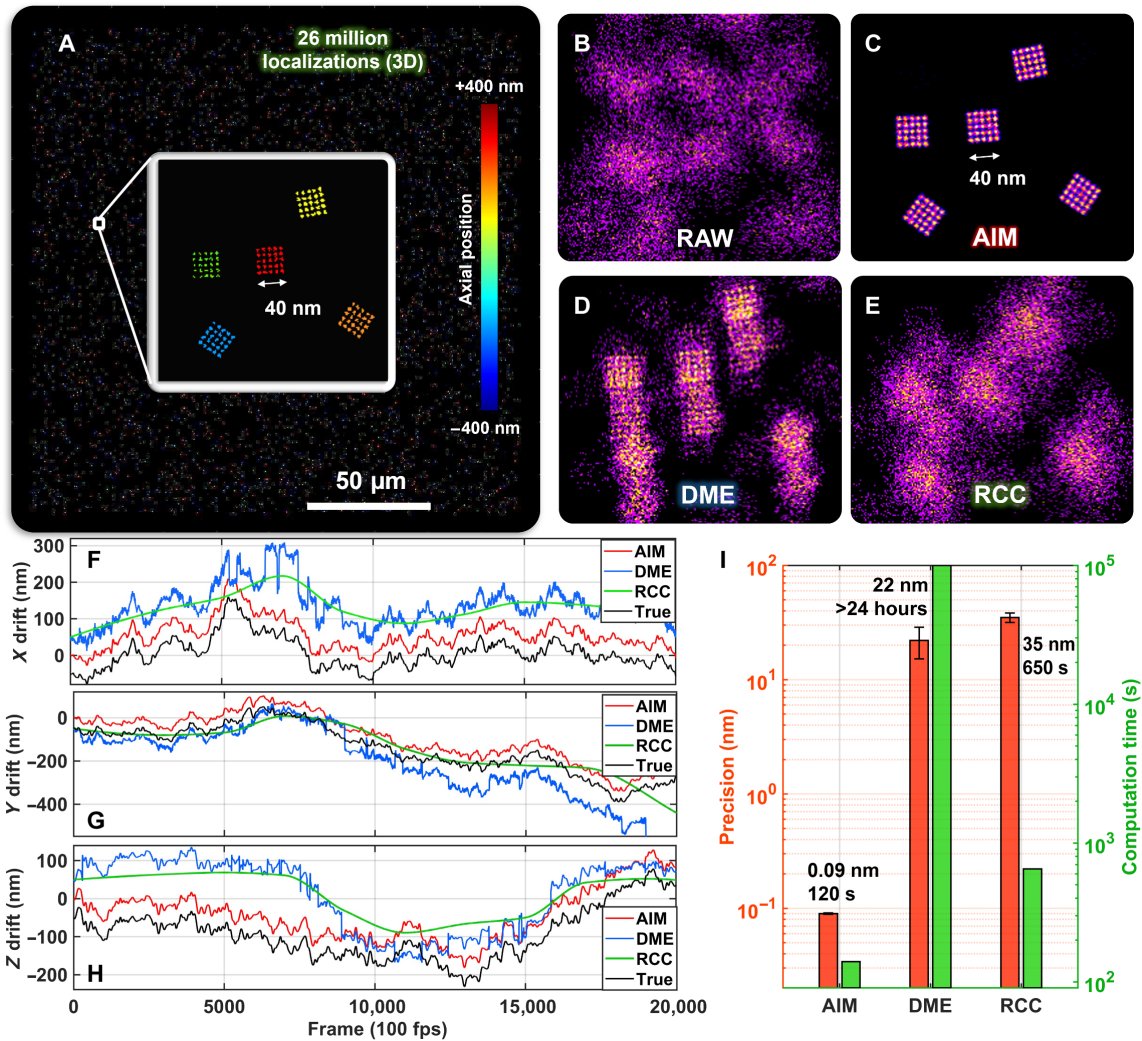
Performance benchmarking using simulated datasets. (**A**) Simulated three-dimensionally distributed DNA origami structures with 25 target sites spaced 10 nm apart in the lateral dimension. (**B** to **E**) The zoomed region without (RAW) and with the three drift correction algorithms (AIM, DME, and RCC). (**F** to **H**) Comparison of the three-dimensional (3D) drift trajectory using AIM, RCC, and DME. It is important to note that any apparent vertical shifts in the trajectories, either upward or downward, have been introduced solely to enhance visual distinction and have no substantive effect on the underlying data. The drift trajectory tracked by AIM is nearly identical to the ground truth trajectory. (**I**) Comparison of drift tracking precision and computation speed for AIM, DME, and RCC. The error bars for computation time are invisible in the figure. Given the camera speed of 100 frame per second, the computation speed of AIM is faster than the data acquisition speed, suggesting that AIM could achieve online drift correction with first-stage estimation. Tracking intervals of AIM, DME, and RCC were 0.2 s (20 frames), 0.02 s (2 frames), and 20 s (2000 frames), respectively.

We first benchmarked the performance of AIM using simulated datasets that mimic a typical dataset captured by high-throughput localization microscopy, which contains 26 million localizations from 20,000 images with an image size of 2048 × 2048 pixels and a pixel size of 100 nm. In comparison with the ground truth, the most commonly used drift correction algorithm, RCC (*8*), and the recently reported state-of-the-art algorithm, DME (*10*), we demonstrated the impact of correcting high-frequency drift (>0.5 Hz) on image resolution. As shown in Fig. 2, our result showed that AIM can precisely track both high-frequency and slow-varying drift (Fig. 2, F to H) with a subsecond tracking interval (0.2 s or 20 frames), which results in a sub-angstrom residual drift error (~0.09 nm). In contrast, for the same dataset, RCC can only use low tracking frequency (every 2000 frames) to track the slow-varying drift, which is mainly limited by the large memory consumption of RCC. As the RCC algorithm estimates the drift by calculating the correlation efficiency map of the temporally segmented super-resolution images, it requires a large amount of random-access memory (RAM) (up to ~40 GB with 10 segments or every 2000 frames used in this study) to cache a series of the temporally segmented super-resolution images with a high-performance computation platform. On the other hand, DME can use a higher tracking frequency (every few frames) to achieve a high tracking precision (*10*) for high-frequency drift. However, because of insufficiently accurate initial estimates from RCC, DME sometimes fails to converge to global minima. Both compromised tracking frequency in RCC and limited robustness in DME result in a larger drift correction error (35 and 22 nm, respectively), making them difficult to identify the ultrafine structures spaced at 10 nm apart. In addition, DME and RCC require a large memory size and long computational time (Fig. 2I) to handle the large localization set generated from the high-throughput localization microscopy. In contrast, AIM is a localization coordinate-driven algorithm, and it only uses small RAM (<4 GB) to store the localization coordinates and is rather computationally efficient for big SMLM datasets without the need for high-end computational resources.

We also evaluated the drift tracking precision and computation speed of AIM in various scenarios (e.g., tracking frequency, emitter density, spatial resolution, and magnitude of drift), as shown in fig. S2. We found that for a high tracking frequency (5 Hz), higher emitter density and spatial resolution help to improve the tracking precision of AIM. However, their influence becomes minimal for lower tracking frequencies. We also note that the tracking precision starts to deteriorate at a very high tracking frequency (10 Hz), due to the reduced number of emitters for each drift subset used in the first-stage drift estimation. On the other hand, the tracking precision is largely affected by a lower tracking frequency when the magnitude of drift is large. At a high tracking frequency (e.g., 2 Hz), the tracking precision remains subnanometer scale regardless of the magnitude of system drift. This result suggests that a high tracking frequency is critical for maintaining a robust performance of drift correction. Last, as the tracking frequency increases, the computation time increases with higher emitter density and tracking frequency. The scenarios within the lines in fig. S2 are those that can achieve computation speed faster than the data acquisition speed at subnanometer precision. It should be noted that for a high tracking frequency of 2 Hz or more, the precision of 0.1 nm can be achieved for moderate emitter density and spatial resolution in SMLM.

### Experimental high-throughput DNA-PAINT imaging of DNA origami

**Fig. 3.**
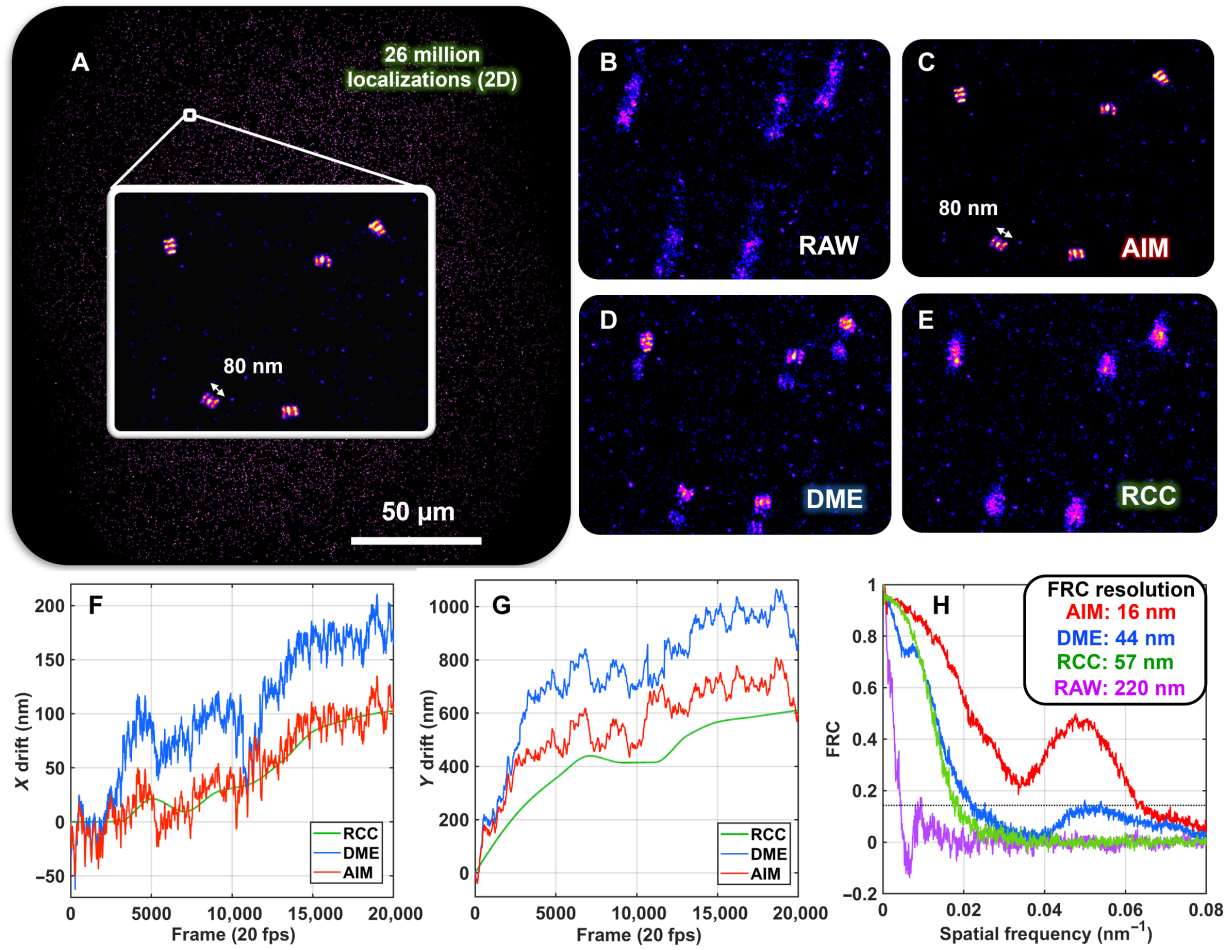
Performance benchmarking using DNA-PAINT imaging of DNA origami. (**A**) DNA origami structure with three target lines spaced at 40 nm apart in the lateral dimension. (**B** to **E**) The zoomed region without and with three drift correction algorithms (AIM, DME, and RCC). (**F** and **G**) Comparison of the 3D drift trajectory retrieved with AIM, RCC, and DME. The computation time for the three algorithms is 140 s (AIM), 350 s (RCC), and 7 hours (DME), respectively. (**H**) FRC resolution of the image corrected by AIM, DME, and RCC, as well as the one without drift correction (RAW). Tracking intervals of AIM, DME, and RCC were 0.2 s (20 frames), 0.02 s (2 frames), and 20 s (2000 frames), respectively.

We next validated the performance of AIM in super-resolution imaging of DNA origami. As shown in Fig. 3, AIM-based drift correction provides an optimized resolution of 16 nm, which is improved by 28 and 41 nm compared to those corrected by DME and RCC, consistent with our simulation results. The tracking interval for RCC and DME is set to be 2000 frames and 2 frames, respectively, limited by the memory of our workstation and from the recommended settings (*8*, *10*). The three-line patterns of DNA origami spaced at 40 nm apart are clearly visible in the image corrected by AIM but not in those corrected by DME or RCC. It is of note that the computation speed for AIM is also the fastest (140 s), especially compared to the computationally intensive DME (~7 hours).

### Experimental high-throughput STORM imaging of cell and tissue section

**Fig. 4.**
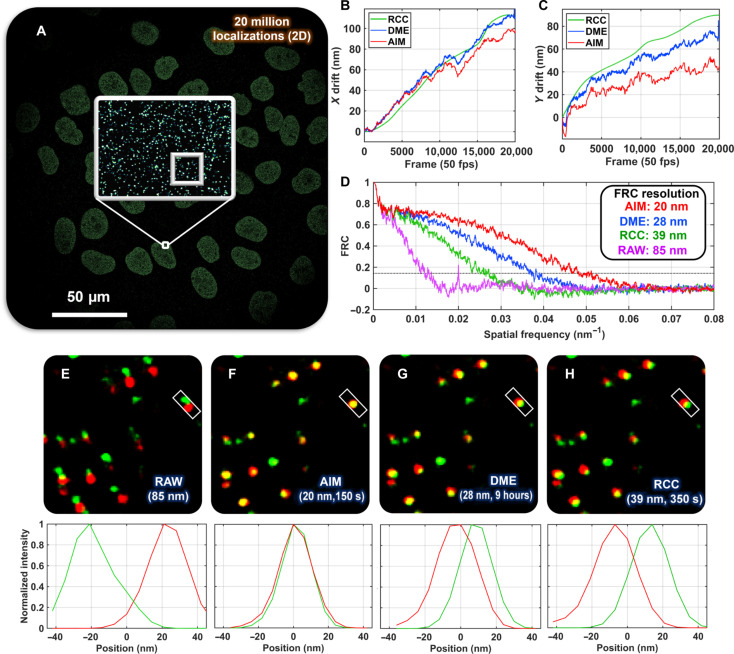
Performance benchmarking using STORM imaging of cell. (**A**) STORM image of CTCF structure on MCF10A cells. (**B** and **C**) Comparison of the 2D drift trajectory retrieved using AIM, RCC, and DME. The computation time for the three algorithms is 150 (AIM), 350 s (RCC), and 9 hours (DME), respectively. (**D**) FRC resolution for the images corrected by AIM, DME, and RCC. For each case (RAW, AIM drift corrected, DME drift corrected, and RCC drift corrected), we divided the entire set of localizations into two parts along the temporal dimension. The first part contains the localizations from the first 9000 frames, while the second part contains the localizations from the remaining 11,000 frames. As the number of localizations decreases during image acquisition, the total number of localizations in the first 9000 frames is similar to that in the subsequent 11,000 frames. The merged super-resolution image of the image reconstructed from the first half localizations in the temporal dimension (green) and the image reconstructed from the second half localizations (red) without drift correction (RAW) (**E**) and with AIM (**F**), DME (**G**), and RCC (**H**) are compared. The global colocalization ratio between the reconstructed STORM images from the first and second half localizations of RAW data without drift correction and drift-corrected data by AIM, DME, and RCC is 6, 52, 35, and 26%, relatively. The below figures illustrate the corresponding colocalization profiles of the cluster in the rectangle box. Tracking intervals of AIM, DME, and RCC were 0.2 s (20 frames), 0.02 s (2 frames), and 20 s (2000 frames), respectively.

We further illustrated the improved image resolution of AIM-based drift correction on biological cells on image resolution of biological cells. Figure 4 shows the reconstructed stochastic optical reconstruction microscopy (STORM) images of CCCTC-binding factor (CTCF) structure in MCF10A cells. AIM-based drift correction improves the Fourier-ring correlation (FRC) resolution of reconstructed STORM images by 8 and 19 nm, compared to DME and RCC approaches, respectively. Given the lack of ground truth in the biological samples, as shown in Fig. 4 (E to H), the STORM image reconstructed from the first half of localizations in the temporal dimension (green) is compared with a second STORM image reconstructed from the second half of localization points (red). Given the same labeled targets, in a drift-free system, these two STORM images should show the highest colocalization rate. As shown in Fig. 4 (F to H), the two STORM images with AIM-based drift correction show the best colocalization with an average colocalization rate of 52%, significantly better than that of DME (35%) and RCC (26%). AIM also achieves the smallest colocalization error (1 nm) compared to those without drift correction (46 nm) and with DME (12 nm) and RCC (21 nm). The colocalization error here is defined as the relative distance between the two clusters. The global colocalization ratio is defined as the number of co-occurrences of localizations from two channels within a distance less than the localization precision, divided by the total number of localizations. This result further demonstrated the improved resolution for AIM together with its computational efficiency (150 s), over other drift correction methods (DME, 9 hours) and RCC (350 s).

**Fig. 5.**
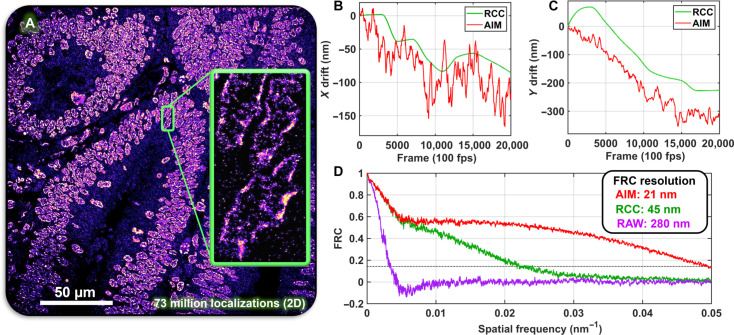
Performance benchmarking using STORM imaging of tissue section. (**A**) STORM image of heterochromatin (immunofluorescently labeled against H3K9me3) in a colon tissue section. (**B** and **C**) Comparison of the 2D drift trajectory retrieved using AIM and RCC. (**D**) FRC resolution of the image that corrected by AIM and RCC. Here, the computation time of DME exceeded the maximum affordable time (>50 hours), which was not shown here. Tracking intervals of AIM and RCC were 0.5 s (50 frames) and 20 s (2000 frames), respectively. The computation time of AIM and RCC were 150 and 350 s, respectively.

Figure 5 shows the super-resolution image of heterochromatin from a colon tissue section after AIM-based drift correction. AIM-based drift correction improves the image resolution defined by FRC by 24 nm (from 45 to 21 nm), compared to RCC. The recovered system drift trajectory by AIM clearly shows the presence of both high-frequency and low-frequency drift, while RCC shows only the recovered low-frequency drift trajectory.

**Fig. 6.**
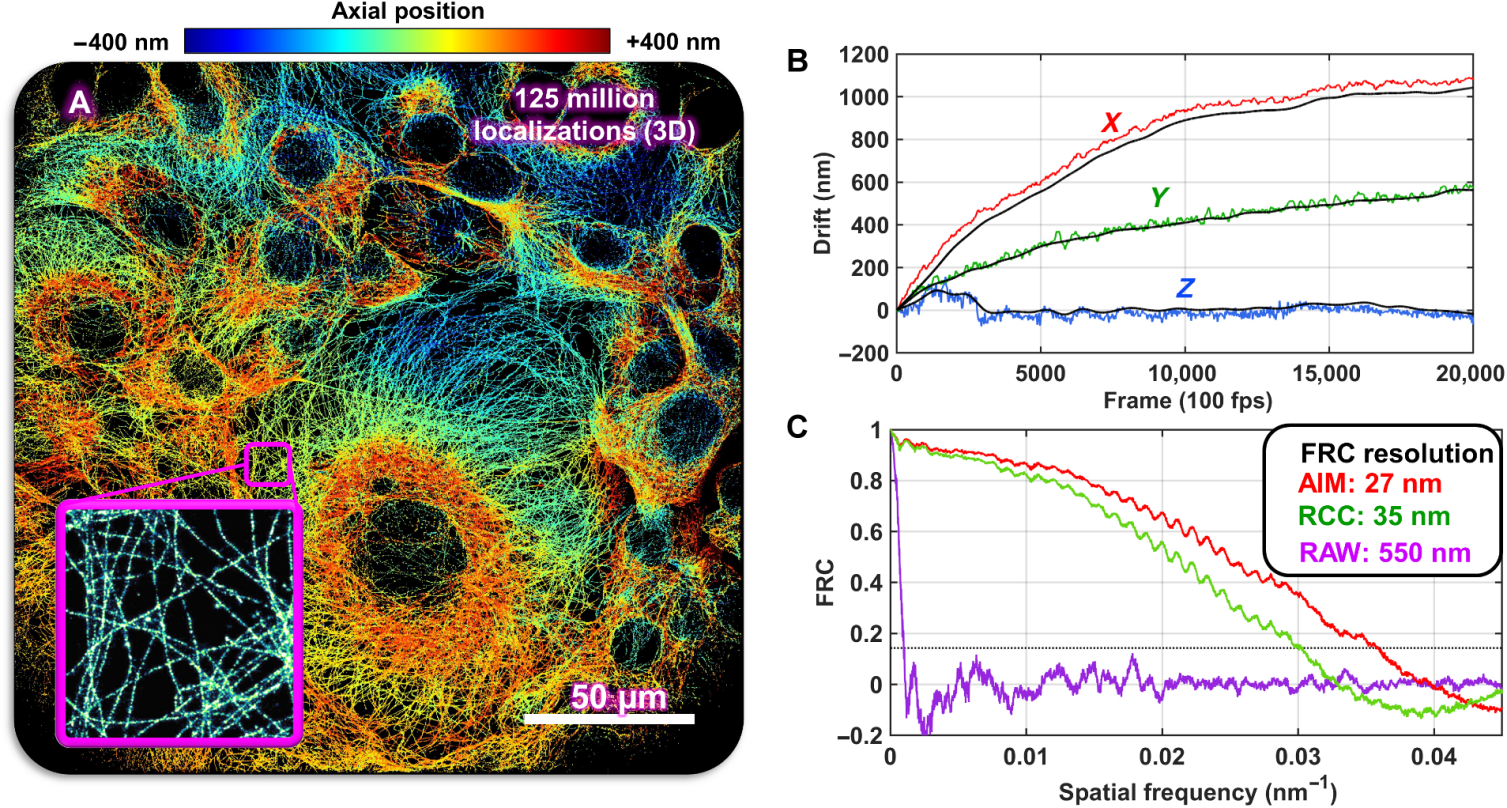
Performance benchmarking using 3D STORM imaging of cell. (**A**) 3D STORM image of microtubules in COS-7 cells. (**B**) Comparison of the 3D drift trajectory retrieved using AIM and RCC (black line). (**C**) FRC resolution of the image that corrected by AIM and RCC. Here, given the computation time of DME exceeded the maximum affordable time (50 hours), the result was not included here. Tracking intervals of AIM and RCC were 0.5 s (50 frames) and 20 s (2000 frames), respectively. The computation time of AIM and RCC were 300 and 650 s, respectively.

AIM can also be applied in three-dimensional (3D) high-throughput SMLM images. As shown in Fig. 6, by correcting both high-frequency and low-frequency drift, AIM improves the image resolution by 8 nm from 35 to 27 nm. Note that DME cannot handle large datasets with more than 30 million localizations (data used for Figs. 5 and 6) on our computer due to its high computational complexity. AIM uses a standard consumer-level computer and does not require a high-end computation platform (e.g., graphics processing unit (GPU) and high memory requirement) to achieve this performance. Therefore, AIM meets the big-data demand for a high-throughput high-content SMLM system with a large imaging field of view (FOV) that generates hundreds of millions of molecules in a single SMLM data (*16*–*18*).

## DISCUSSION

In this study, we demonstrated that our AIM algorithm is highly efficient and accurate in eliminating drift-induced resolution degradation in SMLM without the need for substantial computational resources. Conventional marker-free postprocessing drift correction methods require rather large memory and long computation time, which are impractical to process large datasets at a high tracking frequency. The results of RCC shown in our benchmarking are based on reducing the tracking frequency, which is one of the compromised solutions to handle the high-throughput dataset. It is feasible to reconfigure the RCC algorithm to accommodate a higher tracking frequency (e.g., 20 to 100 frames) for large datasets by discarding the intermediate images at each step, thereby addressing the issue of limited memory to achieve a tracking precision of several nanometers. However, even if the computer memory allows a higher tracking frequency, the extensive calculation of cross-correlations up to millions of times for such a large dataset would considerably increase the computational time, extending it to a span of days to months, rendering it impractical for routine data processing. Furthermore, this approach requires a high level of user expertise to efficiently modify the original RCC algorithm, introducing an additional layer of complexity to its application.

An alternative strategy is to select a small ROI for drift correction. However, this strategy introduces new challenges for non-expert users as it requires proper selection of ROIs with sufficient localizations, which is functionally unavailable for existing RCC and DME. Since biological samples are often heterogeneous, selecting small ROIs can result in varying levels of precision. In addition, it only uses partial information from the entire dataset and achieves a compromised precision. To evaluate whether smaller ROIs reduce the drift correction precision, we simulated small SMLM datasets with an image size of 128 × 128 pixels (15 emitters per frame, 20,000 frames, and 300,000 localizations) for drift tracking intervals ranging from 20 to 500 frames.

As shown in fig. S3, for the same partial dataset and tracking frequencies, AIM still achieves the best tracking precision (~1.2 nm) with approximately two to three times improvement in all three dimensions compared to DME and RCC, at a computational speed with two to three orders of magnitude faster. The substantially improved precision (<0.1 nm) of AIM from the full high-throughput SMLM dataset justifies that selecting a smaller ROI from a large dataset increases the tracking error due to the information loss. It is feasible to achieve acceptable precision by carefully selecting ROI size and tuning parameters for a large dataset, as shown in fig. S4. Alternatively, a full large dataset can be partitioned into multiple smaller subsets spatially or temporally, followed by averaging or connecting the drift trajectories estimated in each subset, as shown in figs. S5 and S6. These results suggest that using the full information is not merely averaging or connecting the partitioned drift trajectories. Using the entire FOV remains the simplest and most efficient way to achieve state-of-the-art precision and robustness.

While conventional drift correction methods estimate the drift based on the temporally segmented partial localization datasets, the improved accuracy of AIM comes from its ability to fully use the entire large dataset without information loss. AIM introduces a two-stage processing strategy that uses the entire drift-corrected localization dataset obtained at the first stage as a new reference for a second, more refined stage of drift correction that results in improved robustness and precision. A large dataset makes it even more powerful to achieve unprecedented precision down to the angstrom scale (Fig. 2 and fig. S1).

An ideal algorithm should be robust to achieve optimal performance for datasets under various scenarios without extensive manual parameter tuning. AIM achieves optimal results for a wide range of simulation and experimental datasets. We initially demonstrated the performance of AIM under various simulation conditions, including emitter density, spatial resolution, and magnitude of drift. Figure S2 illustrates that AIM can achieve subnanometer precision over a tracking interval of 10 to 50 frames, with optimal precision at a tracking interval of 20 frames. Subsequently, we applied the identical parameter settings (at a tracking interval of 20 frames) in both simulation (Fig. 2) and biological experiments conducted under a range of imaging conditions (Figs. 3 to 6), and AIM consistently shows superior performance. The recently reported DME algorithm shows improved accuracy, especially for high-frequency drift, when its numerous parameters are properly tuned. However, when the drift is minimal, it could introduce high-frequency drift artifacts (fig. S7). Users often need to compare tracking precision or FRC resolution across a range of tracking intervals for each dataset to find the optimal value that yields the best image quality. Otherwise, DME can converge to local minima and result in substantial tracking errors, making it challenging to achieve robust performance for a wide range of datasets. Furthermore, DME is a computationally intensive iterative algorithm that is rather slow and can take days to handle one large high-throughput imaging dataset acquired in minutes, making the parameter tuning even more challenging.

The high level of computational efficiency is another major advantage of AIM. It uses localization coordinates that do not involve large image-based processing and thus can perform ultrafast drift tracking without high-performance computing platforms. It only requires a limited memory (<4 GB depending on the size of the localization dataset) to store the localization coordinates. AIM estimates the drift by simply counting the number of intersected localizations, which is highly efficient without the need for GPU acceleration. It can achieve real-time drift tracking for high-throughput SMLM datasets. AIM is also model free, suggesting that it can robustly track drift with minimal influence from various imaging conditions. Our simulation and experimental results demonstrate that AIM can achieve subnanometer drift correction precision, which is comparable to the state-of-the-art marker-assisted approaches. These advantages make AIM a robust marker-free drift correction approach as a perfect alternative to marker-assisted approaches. Further, although this study focuses on datasets from STORM and DNA points accumulation for imaging in nanoscale topography (DNA-PAINT) based SMLM, this approach in principle could be extended to drift correction for other localization-based super-resolution imaging techniques such as DNA-PAINT minimal fluorescence photon fluxes nanoscopy (*19*).

In this study, we report a marker-free and model-free method, named the AIM, for 3D high-frequency drift correction based on the localization coordinates. AIM reduces the drift error to 0.1 nm and enables real-time drift tracking for high-throughput SMLM. We demonstrated that AIM can substantially reduce drift error and enhance the spatial resolution of SMLM compared to existing state-of-the-art marker-free drift correction algorithms. Our results underline the importance of correcting the high-frequency drift commonly present in SMLM systems to achieve the optimal image resolution. Furthermore, we offer AIM as freely available software, designed for broad compatibility with existing SMLM datasets to instantly boost its image resolution.

## MATERIALS AND METHODS

### Algorithm of AIM

For SMLM, the spatial drift can be considered as a 3D translational motion (*8*, *10*). A super-resolution image consists of a set of localized fluorescent emitter coordinates *C* = {*p*(*x*_1_, *y*_1_, *z*_1_, *t*_1_), …*p*(*x_m_*, *y_m_*, *z_m_*, *t_m_*)} acquired at different time throughout the entire imaging process, where *x*, *y*, and *z* are each emitter’s spatial coordinates, *t* is the emitter’s temporal position, and *m* is the total number of localizations for the entire dataset. In a drift-free system, those localized emitters originating from the same sets of emitters should “intersect” within a threshold distance *D* determined by localization precision (σ) (Fig. 1). Drift reduces the number of intersected emitters. By maximizing the number of intersected emitters between the temporally separated segments, their relative shift can be estimated. However, because each temporal segment only contains a small portion of all localized points (*m*/*n*), the estimated drift often lacks sufficient accuracy. AIM uses a two-stage processing. The initial stage estimates a preliminary drift to compensate the entire dataset, which is used as a new reference for a second, more refined stage of drift estimation. During this second phase, the now drift-corrected reference set, which retains the complete dataset information, facilitates the identification of the maximal number of intersected localizations. This process allows for enhanced precision and robustness in the drift estimation. The detailed procedures are described below.

1) We first partition all localized emitters (*C*) into *n* temporally segmented subsets (*C*_0_, *C*_1_… *C*_*n*−1_) where each temporal subset contains a specified number of image frames with approximately 1/*n* of all localized emitters, acquired within a certain temporal interval *T* (e.g., 20 image frames at an exposure time of 10 ms within a time interval *T* = 0.01 ×20 = 0.2 s). The first (initial) temporal subset coordinate (*C*_0_) is referred to as the reference subset (*C*_*r*_), and the subsequent subsets (*C*_1_, *C*_2_… *C*_*n*−1_) are denoted as the drift subsets.

2) Next, we describe how to identify the relative drift between the reference subset (i.e., *C*_*r*_ = *C*_0_) and a drift subset *C*_*d*_ (e.g., *C*_*d*_ = *C*_1_ for the first drift subset), by calculating the peak shift position on an intersected shift-map [*I*(*d*)]. When the distance of the localized coordinate pairs (*d*) from the reference subset and the shifted subset is less than a threshold *D* (e.g., 20 nm), this pair is considered as intersected. The intersected shift-map is created by calculating the number of the intersected coordinate pairs between the reference subset (*C*_*r*_) and a shifted subset (*C*_*s*_), denoted as *I*(*d*) = *n*(*C*_*r*_ ⋂ *C*_*s*_). To create a shifted subset (*C*_*s*_), the coordinates of the drift subset *C*_*d*_ are shifted with a step size of *D* in a local region with a radius of *R*, where *R* should be larger than the maximum drift between two adjacent subsets (e.g., 60 nm). Then, the peak position with shift vector $\vec{S_{i}}=\left(d_{ix},d_{iy},d_{iz}\right)$ is calculated on the intersection shift-map via the computationally simple and fast Fourier harmonic analysis (*20*) to determine the drift.

3) For the next subset (*C*_*i*+1_) (e.g., *C*_2_), the previously determined drift $\vec{S_{i}}$ is corrected to obtain an adaptively drift-corrected subset ($C_{d}=C_{i+1}-\vec{S_{i}}$), which is used as an updated *C_d_* to find the shift vector $\vec{S_{i+1}}$ for the next temporal subset as described in step 2. Here, instead of finding the relative shift between the reference set and all of the temporal subsets, we use an adaptively updated drift-corrected subset *C*_*d*_ from *C*_*i*+1_ based on the drift estimation from the prior subset *C*_*i*_ to find the shift vector ($\vec{S_{i+1}}$) for the next adjacent temporal subset (*C*_*i*+1_). Thus, drift tracking for each subset (*C*_*i*+1_) is equivalent to measuring its relative drift by referencing its prior subset (*C*_*i*_). This adaptive approach has two key advantages. First, it transforms the long time interval drift into short time interval drift relative to the adjacent temporal subset that often has a small drift distance, thus substantially reducing the searching region of intersection shift-map. Second, it can largely reject the false-positive peaks of the intersection shift-map, thus enhancing the robustness of the drift estimation. The same process is repeated for the rest of the drift subsets (e.g., *C*_3_,…*C*_*n*−1_).

4) On the basis of the estimated discrete drift positions at each time interval *T*, the time points within each interval *T* can be estimated by cubic spline interpolation. Then, we can subtract the estimated drift positions at each image frame to get the drift-corrected localization dataset *C*_*dc*_. The first stage drift correction is composed of steps (1 to 4). As its calculation is based on a comparison of the temporally separated subset with partial localizations (*m*/*n*), the intersection shift-map usually has a limited signal-to-noise ratio, thus reducing the precision for drift estimation.

5) To achieve an improved precision, we apply a second-stage drift correction by taking advantage of the full dataset. The entire drift-compensated dataset *C*_*dc*_ determined above in the first stage is updated as the new reference (*C*_*r*_ = *C*_*dc*_) used in the second stage. We then again partition the drift-compensated localization dataset *C*_*dc*_ into *n* subsets as the drift subsets (*C*_*dc*1_, *C*_*dc*2_… *C*_*dcn*_) at the same temporal interval *T* as stage 1 and repeat steps (2 to 4) as described above to precisely estimate the residual drift for the entire dataset. As the second stage uses a new reference set (roughly corrected reference set) that now contains all localizations (*m*), the robustness and precision for the second-stage drift estimation are much improved.

### High-throughput SMLM imaging

The instrument setup for the high-throughput SMLM system is shown in fig. S8. A high-power 638-nm excitation laser (2 W) was used as the light source, coupled into a fast-vibrating multimode fiber (M97L02, Thorlabs) to generate flat-field illumination. A TIRF objective lens (UPlanApo 60×/1.50 oil HR, Olympus) was used for DNA-PAINT imaging, and an oil-immersion objective lens (UPlanXApo 60×/1.42 oil, Olympus) was used for STORM imaging experiments. The sample was mounted on a three-axis high-precision piezo motor linear stages (Agilis Piezo Motor series, Newport) with a travel distance of 12 mm for both lateral and axial movement. The fluorescence signals were collected by scientific complementary metal-oxide semiconductor cameras (Hamamatsu ORCA-Flash4.0). The pixel size corresponding to the sample plane was ~100 nm, thus providing a FOV of ~200 μm by 200 μm. In addition, an 850-nm laser (CPS850S, Thorlabs) was used as the light source coupled with the linear CCD (LC100, Thorlabs) and piezo focusing stage (Nano-F100, MadCityLabs) for focus locking (*21*). We used the redundant array of independent disks storage system (LaCie 12big Thunderbolt 3) for high-speed acquisition and storage of large image data. We used an illumination power intensity of ~4 kW/cm^2^ for all DNA-PAINT and STORM imaging experiments in this study. For the DNA-PAINT imaging experiment (Fig. 3), an exposure time of 50 ms was used, and for other STORM imaging experiments of cells and tissue sections, an exposure time of 20 ms was used for each frame.

### Numerical simulation

The simulated data shown in Fig. 2 were generated to mimic DNA origami structures with 25 target sites spaced 10 nm apart in the lateral dimension, distributed in three dimensions. The DNA origami structures were distributed with an axial position range from −400 to 400 nm with a random uniform distribution. The blinking events for each molecule follow a uniform random distribution with an on/off ratio of 1:120. We quantified tracking precision and computation time under different tracking frequencies for various imaging conditions including localization density, localization precision, and the magnitude of system drift. The selection of these imaging parameters is justified below.

#### Emitter density

The commonly used emitter density for SMLM imaging is ~0.1 emitters/μm^2^, which is a good compromise between imaging speed and spatial sparsity important for spatial resolution. For scenarios where the sample covers most areas within the FOV, each dataset included ~90 million localizations in our simulation. Considering the entire FOV may not be always fulfilled, we also simulated two other localization densities (0.01 and 0.05 localizations/μm^2^), corresponding to sample occupancy ratios of 10 and 50%, respectively.

#### Spatial resolution

The spatial resolution of SMLM varies depending on the brightness of the labeled fluorophores. Dim fluorophores (e.g., fluorescent proteins used in photo-activated localization microscopy) achieve a spatial resolution of 30 to 50 nm, while bright fluorophores (e.g., bright organic fluorophores used in STORM and DNA-PAINT) give a resolution of 10 to 30 nm. Therefore, we simulated three spatial resolutions (10, 20, and 40 nm) to evaluate the performance of our algorithm.

#### Magnitude of drift

The magnitude of drift for the imaging system largely depends on the imaging environment. In a stable environment where the optical microscope sits on an optical table with active vibration isolation in a room with constant temperature, the system drift can be minimized to several nanometers per second. As the image resolution of the SMLM system can degrade with the system drift in the long period of image acquisition, we simulated three different magnitudes of system drift, with a root mean square drift of 5, 10, and 20 nm/s, respectively. The drift was simulated as the added noise with a cumulative normal distribution, as described in the previous study (*10*).

We generated Fig. 2 and fig. S1 using 10 randomly simulated datasets. The simulated datasets consist of 20,000 frames of single-molecule images for a FOV of 2048 by 2048 pixels per image to mimic a typical dataset captured by a high-throughput localization microscopy system. The datasets were simulated with an emitter density of 0.03 emitters/μm^2^, a spatial resolution of 5 nm, and a root mean square drift of 20 nm/s. In fig. S3, we simulated 10 datasets that contain 20,000 frames of single-molecule images with a FOV of 128 by 128 pixels per image. The datasets were simulated with an emitter density of 0.1 emitters/μm^2^, a spatial resolution of 40 nm, and a root mean square drift of 10 nm/s to mimic small SMLM datasets with small FOV (12 μm by 12 μm), small number of localizations (15 localizations per frame), and standard resolution (40 nm).

### Performance benchmarking

We benchmarked the performance of AIM by comparing it with two state-of-the-art drift correction methods including one of the most commonly used RCC (*8*) and the recently reported state-of-the-art DME (*10*). A workstation (Dell) equipped with a multicore central processing unit (CPU) (Intel Core i7-11700) and 64 GB of RAM was used for high-speed data processing. For fair comparison results, we performed different algorithms on the same computer with MATLAB R2020 using CPU devices. The drift tracking precision in this study is defined as the SD of the tracking error between the estimated drift and the ground truth value. For the high-throughput SMLM dataset with an image size of 2048 by 2048 pixels, we used 20 frames per bin for AIM, 2000 frames per bin for RCC, and 2 frames per bin for DME. The number of frames per bin for RCC was limited by the size of the memory available on the computer. RCC and DME used approximately 40GB of memory in the computation process. Our computer cannot handle a large dataset with over 30 million localizations for DME. Both the pixel size of the cross-correlation map used in RCC and the intersection distance used in AIM were set to 20 nm.

For single-molecule localization, our previously reported algorithm (*20*, *22*, *23*) was used to achieve a computation speed of ~2 million localizations/s on the same computer as described above. To calculate FRC resolution (*24*), we evenly split the entire localization dataset into two subsets along the temporal dimension, the two super-resolution images reconstructed using each subset were used to calculate the FRC resolution.

### Design, self-assembly, and purification of DNA origami

The design of DNA origami structures was done using the design module of Picasso (*25*). Backbone staples, docking staples, and biotinylated staples were purchased from Integrated DNA Technologies, Inc. The DNA origami structures were self-assembled in one reaction mixture in 1× Tris-EDTA buffer supplemented with 12.5 mM MgCl_2_. The components included 10 nM M13 bacteriophage single-stranded DNA scaffold p7249 (New England BioLabs, N4040S), 250 nM folding staples, 250 nM biotinylated staples, and 250 nM DNA-PAINT docking strands. The mixture was heated at 80°C for 5 min, followed by 65°C for 15 min to denature scaffold and staples strands before being subjected to a thermal gradient from 60° to 4°C (3 min 12 s/°C) using a thermocycler (Bio-Rad, T100TM Thermal Cycler) for annealing these staples strands to their complementary segments on the scaffold, resulting in folding the DNA origami into the designed shape.

The self-assembled DNA origami mixtures were ultrafiltrated using small-scale molecular weight cutoff membranes for damage-free, native purification, and enrichment of DNA origami from excess strands (*26*). Briefly, the reaction mixtures were added into the Amicon Ultra 0.5-ml filters with 50-kDa molecular cutoff membranes (Merck/EMD Millipore, UCF505096), spun for three times at 10,000 relative centrifugal force, 5 min each in 1× FoB5 buffer (5 mM tris base, 1 mM EDTA, 5 mM MgCl_2_, and 5 mM NaCl). The purified and concentrated DNA origami were collected and stored at −20°C.

### Sample preparation for imaging DNA origami structure

To prepare DNA origami for imaging, a coverslip (Thermo Fisher Scientific, no. 1.5, 22 mm by 22 mm, 12541B) as the substrate was attached to another piece of cover glass (Corning, no. 1.0, 18 mm by 18 mm, 2845-18) via double-sided tape to create a ~20-μl flow chamber. In our chamber, a 20 μl of biotinylated bovine albumin (1 mg/ml; Sigma-Aldrich, A4503) in buffer A [10 mM tris-HCl, 100 mM NaCl, and 0.05% (v/v) Tween 20 at pH 8.0] were incubated for 5 min and then washed with 100 μl of buffer A. Then, 20 μl of streptavidin (0.5 mg/ml; Thermo Fisher Scientific, S888) in buffer A flowed into the chamber and then incubated for 5 min for biotin-streptavidin interaction. After coating the coverslip with biotin-streptavidin, the chamber was washed with 100 μl of buffer A, followed by 100 μl of buffer B [5 mM tris-HCl, 10 mM MgCl_2_, 1 mM EDTA, and 0.05% (v/v) Tween 20 at pH 8.0] before adding purified DNA origami structures in buffer B for 10 min of incubation. A 100 μl of buffer B was then flown into the chamber for final washing. Nanodiamond (Adámas Nano, NDNV140nmPMO) at 1/10,000 dilution in buffer B was added into the chamber and incubated for 10 min as fiducial markers for drift correction in SMLM. Last, the chamber was filled with an imaging buffer containing 5 nM ATTO647N-labeled P1 imager (IDT) in buffer B (*25*) and then sealed with epoxy (Gorilla Glue Epoxy, 5 min). The chamber was stored at 4°C until imaging.

### Immunofluorescence staining for cultured cells and tissue section

For immunofluorescence staining, cells were fixed with 4% paraformaldehyde for 15 min and permeabilized with 0.2% Triton X-100 for 10 min. For the tissue section, the 3-μm-thick tissue sections from formalin-fixed paraffin-embedded (FFPE) tissue blocks were mounted on the poly-d-lysine–coated coverslips, deparaffinized in xylene, and rehydrated in graded ethanol and lastly in distilled water. The antigen retrieval was performed as previously described (*27*). The samples were incubated with primary antibodies (CTCF, #ab70303; H3K9me3, #ab5408, Abcam) diluted to optimized concentration at 4°C overnight. Alexa Fluor 647–conjugated goat anti-rabbit secondary antibody was applied to the sample at room temperature for 2 hours in the dark. The samples was washed again three times with washing buffer and once with phosphate-buffered saline (PBS) for 5 min per wash and stored in PBS before imaging. The imaging buffer was added into the sample dish right before imaging.

### STORM imaging buffer

STORM imaging buffer for cultured cells contains 10% (w/v) glucose (Sigma-Aldrich), glucose oxidase (0.56 mg/ml; Sigma-Aldrich), catalase (0.17 mg/ml; Sigma-Aldrich), and 0.14 M 2-mercaptoethanol (βME, Sigma-Aldrich). For the formalin-fixed paraffin-embedded (FFPE) tissue section, to reduce the high background caused by the strong scattering of pathological tissue, an optical clearing process was conducted before imaging by immersing the sample in 60% (v/v) 2,2′-thiodiethanol (TDE) for 20 to 30 min to make the sample transparent (*27*). For the STORM imaging buffer of FFPE tissue section, 60% (v/v) TDE solution was used instead of water to match the tissue’s index and contains 10% (w/v) glucose (Sigma-Aldrich), glucose oxidase (0.56 mg/ml; Sigma-Aldrich), catalase (0.17 mg/ml; Sigma-Aldrich), 0.14 M βME (Sigma-Aldrich), and 0.2 mM cyclooctatetraene (Sigma-Aldrich). The imaging buffer was added to the sample dish right before imaging.

## Supplementary Materials

### This PDF file includes:

Supplementary Notes Figs S1 to S8
