# Supplementary material for: Toward drift-free high-throughput nanoscopy through adaptive intersection maximization
Source: Sci Adv. 2024 May 23;10(21):eadm7765. doi: 10.1126/sciadv.adm7765 (PMC11114195; doi:10.1126/sciadv.adm7765)
Supplement: Supplementary file 1 — Supplementary Notes Figs S1 to S8 [file sciadv.adm7765_sm.pdf]

Science Advances
AAAS

Supplementary Materials for

Toward drift-free high-throughput nanoscopy through adaptive intersection maximization

Hongqiang Ma *et al.*

Corresponding author: Hongqiang Ma, mhq@illinois.edu; Yang Liu, liuy46@illinois.edu

Sci. Adv. **10**, eadm7765 (2024)
DOI: 10.1126/sciadv.adm7765

This PDF file includes:

Supplementary Notes
Figs S1 to S8

Supplementary Notes

Parameter setting for AIM

In our algorithm, three parameters can potentially affect the final drift tracking performance: intersection distance (D), tracking time interval (m), and the radius of the local region (R). However, the impact of these parameters varies.

The intersection distance (D) is primarily determined by the localization precision of the dataset. Typically, its value can be set to around three times the localization precision for optimized performance. However, unlike model-based algorithms, our AIM is quite robust to unoptimized parameters. As shown in Supplementary Figure S2B, AIM can still achieve sub-nanometer tracking precision with a fixed intersection distance (D=20nm) for datasets with resolutions ranging from 10nm to 40nm. In practice, if users have little information about the localization precision of the dataset, they can simply set D to 20nm for near-optimal performance.

The radius of the local region (R) is primarily determined by the magnitude of the drift. Typically, its value can be set to around three times the magnitude of the drift to handle extreme jumps in a short period. Since users may not have access to drift information before drift estimation, we've set a fixed value of 60 nm in practice. This value can handle sudden drifts of up to 120 nm in 0.2 seconds. With R = 60 nm, AIM maintains its state-of-the-art performance for all numerical and biological experiments in this study.

The tracking interval (m) is primarily determined by the number of localizations per frame. Typically, it can be set to 20 to 50 frames for high-throughput datasets with a large field of view (e.g., 2048x2048 pixels), and 50 to 100 frames for datasets with a small field of view (e.g., 128x128 pixels). For users with limited knowledge of localization density, opting for m = 100 frames for most datasets carries minimal risk.

For less experienced users, choosing D=20nm, R=60nm, and m=100 frames should yield satisfactory results across various image sizes and imaging conditions without the need for parameter fine-tuning.

Precision improvement of AIM with a multi-stage processing strategy

The drift correction algorithm generally requires tracking the spatial displacement of objects over time. In the case of SMLM which uses coordinates of localized emitters, we need to break down the entire localized points into a certain number (n) of temporally segmented blocks (n segments) and calculate the relative shift between each temporal segment and the first segment (i.e., reference set). However, because each temporal segment only contains a small portion (1/n) of the localized points in the dataset, the estimated drift can be noisy and lacks sufficient accuracy due to the limited number of intersected localizations between temporal segments. AIM addresses these limitations via a two-stage adaptive processing strategy. The initial stage estimates a preliminary drift to compensate for the entire dataset, which will be used as a new reference for a second, more refined stage of drift estimation. During this second phase, the now drift-corrected reference set, which retains the complete dataset information, facilitates the identification of the maximal number of intersected localizations for improved precision and robustness. A third iteration could potentially further improve the accuracy of AIM, but the gains in accuracy become marginal, not significant enough to justify the doubled computation time. However, users who prioritize faster speed can utilize a single stage, while those requiring more precise tracking precision can opt for three stages.

Computational complexity of AIM

The computational complexity of AIM is O(n), where n is the number of localizations, while the computational complexity of redundant cross correlation (RCC) is O(m^2), where m is the image size of the super-resolution image used for cross correlation.

Supplementary Figures

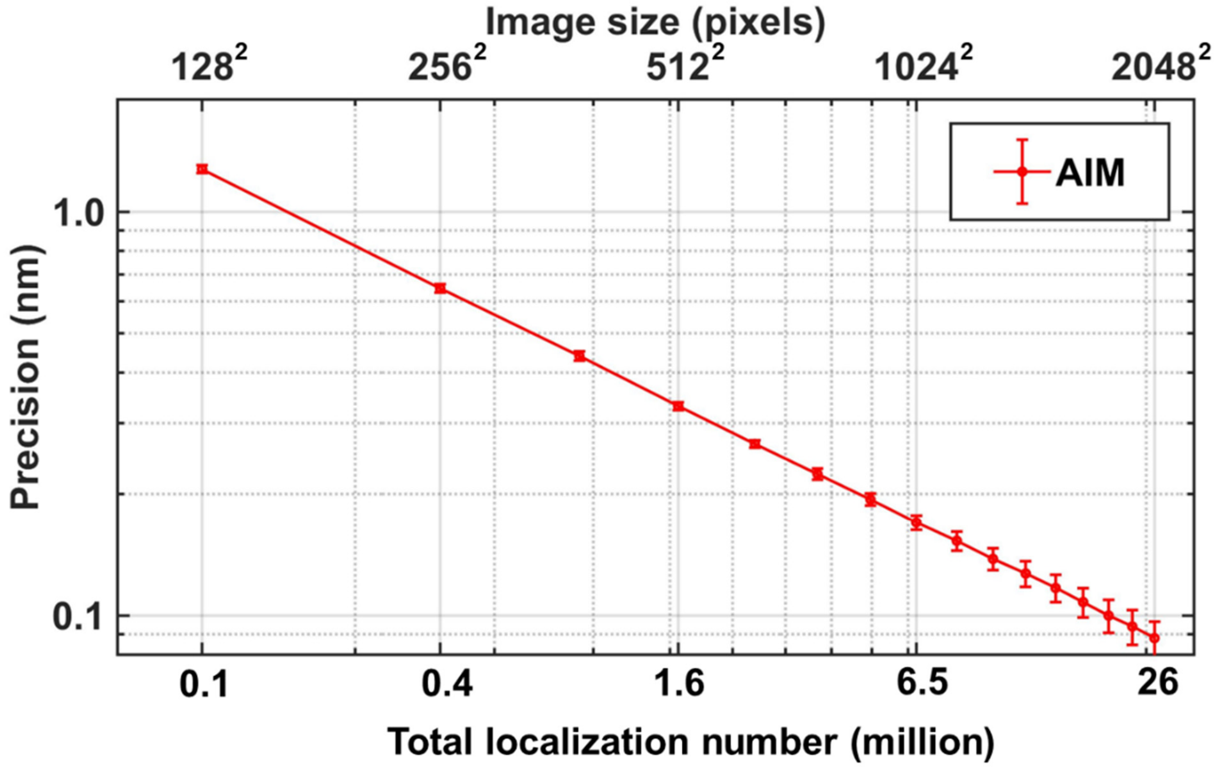

Fig. S1. **Drift tracking precision under a wide range of image sizes and localization numbers.** Datasets are simulated with an image size from 128×128 pixels to 2048×2048 pixels (top x-axis), following the rule of $(128\times n)^2$, where (n=1:16) and a total number of localizations from 0.1 million to 26 million (bottom x-axis). The datasets were simulated with an emitter density of 0.03 emitters/μm^2 (assuming a uniform distribution of emitters across the entire field of view (FOV)), spatial resolution of 20 nm, and root mean square drift of 20 nm/s. The tracking interval for AIM is 20 frames. We performed 10 numerical simulations for each image size and measured the mean value and robustness (represented by the standard deviation) of drift-tracking precision derived from AIM. In principle, drift tracking in SMLM involves using the "intersected" localized emitters across temporal subsets to derive an estimation of their relative shift, and the precision for drift estimation is proportional to $\sigma/\sqrt{N}$, where σ is the localization error and N is the number of intersected localized emitters between temporal subsets. In SMLM, molecules exhibit random blinking behavior across the FOV. By opting for a larger FOV, we can incorporate a greater number of molecules within each temporal subset, thereby accumulating a more substantial pool of intersected molecules. This accumulation facilitates the precise determination of joint positions ($\sigma/\sqrt{N}$). This simulation results align well with the theoretical expectation that the precision of drift tracking improves as the image size increases, or as the number of intersected localizations increases, due to the utilization of greater information content.

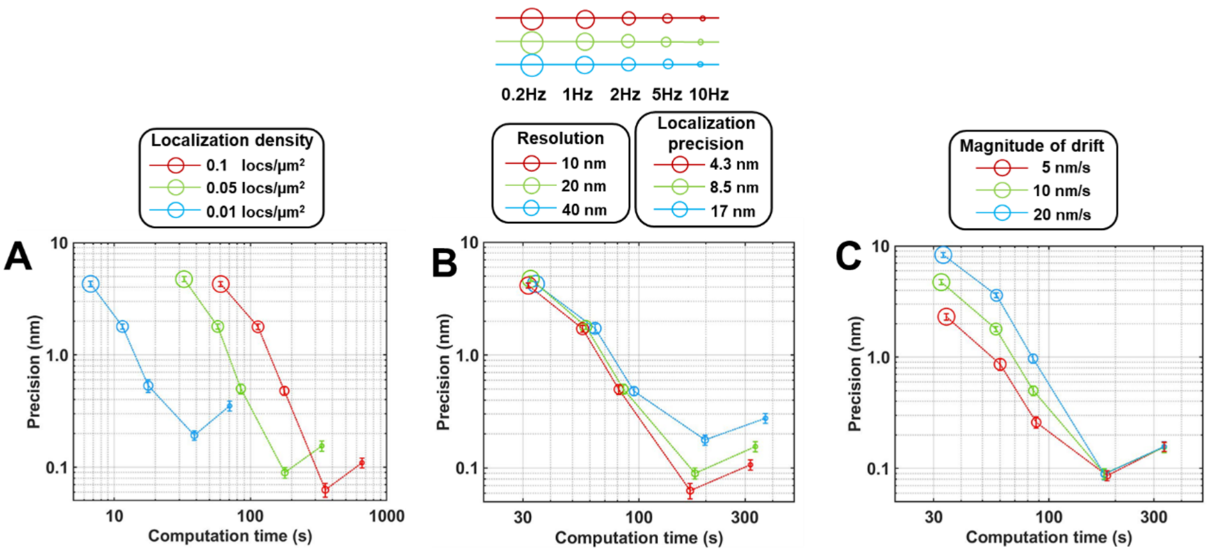

Fig. S2. Drift tracking precision and computational speed of AIM under various imaging conditions. We simulated a wide range of localization density (**A**), spatial resolution/localization precision (**B**), and magnitude of drift (**C**). We performed drift tracking with different tracking intervals (5s, 1s, 0.5s, 0.2s, and 0.1s) corresponding to a frame number of 500, 100, 50, 20, and 10 in each temporal subset, respectively.

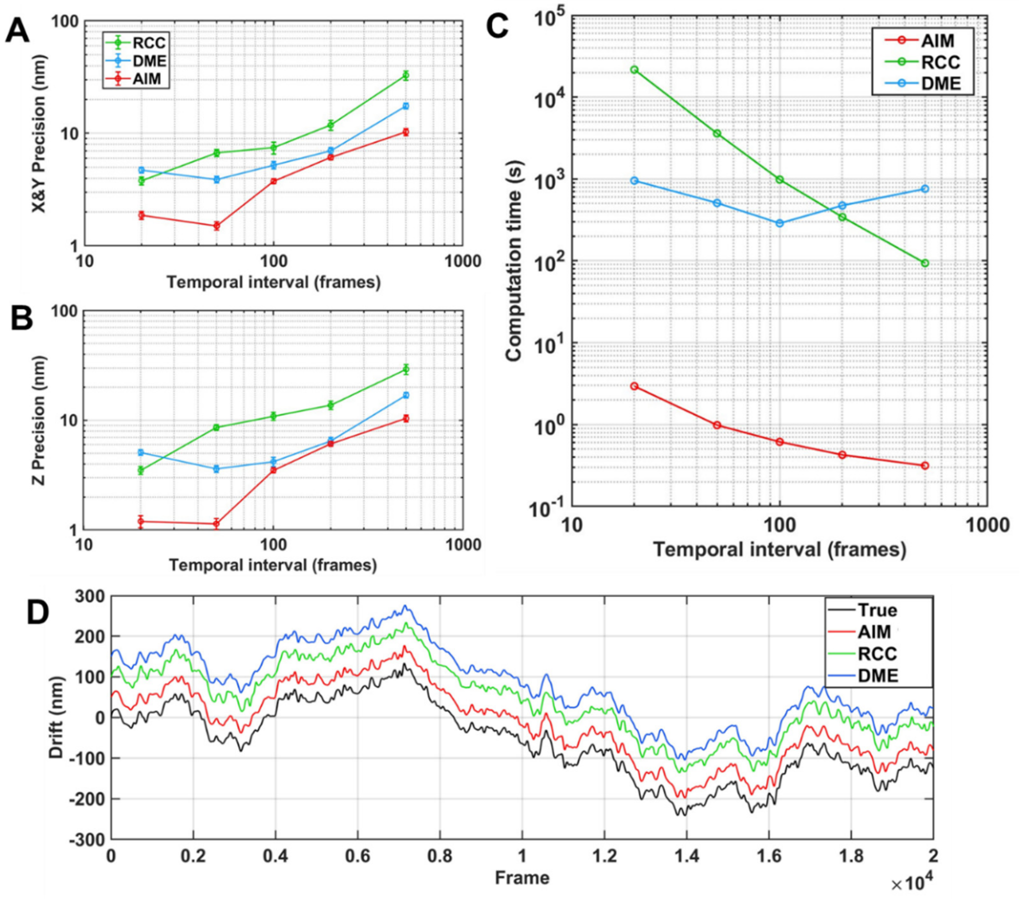

Fig. S3. Performance benchmarking of AIM, DME, and RCC using simulated datasets with a small image size (128×128 pixels). (**A-B**) Drift tracking precision in X, Y, and Z dimensions, (**c**) computation time of AIM, DME, and RCC for different tracking intervals given small SMLM datasets with a size of 128×128 pixels per image. (**D**) Representative drift trajectories for the same dataset that estimated by AIM, DME, and RCC at a tracking interval of 50 frames. The dataset contains 0.1 emitters/μm^2, a spatial resolution of 40 nm, and a root mean square drift of 10 nm/s to mimic small SMLM datasets with a small field-of-view (12×12 μm^2), a small number of localizations (15 localizations/frame). Ten numerical simulations were used to measure the drift-tracking precision of the three algorithms. AIM achieves the best tracking precision of ~1.2 nm in all three dimensions, compared to RCC (tracking precision = 7.5 nm) and DME (tracking precision = 3.9 nm), at a speed of 2-3 orders of magnitude faster than RCC and DME at a tracking interval of 50 frames. This result shows that with the same input dataset, the performance of AIM is also superior to the existing methods (DME and RCC). For model-based algorithm DME, a precise initial estimation with RCC is critical to achieve robust drift tracking results and minimize drift artifacts (jitter). We tested the drift tracking performance of DME with different initial estimations from RCC (200 frames/bin to 2000 frames/bin) and chose the initial estimation of RCC with 500 frames/bin by balancing the drift tracking precision and computation speed.

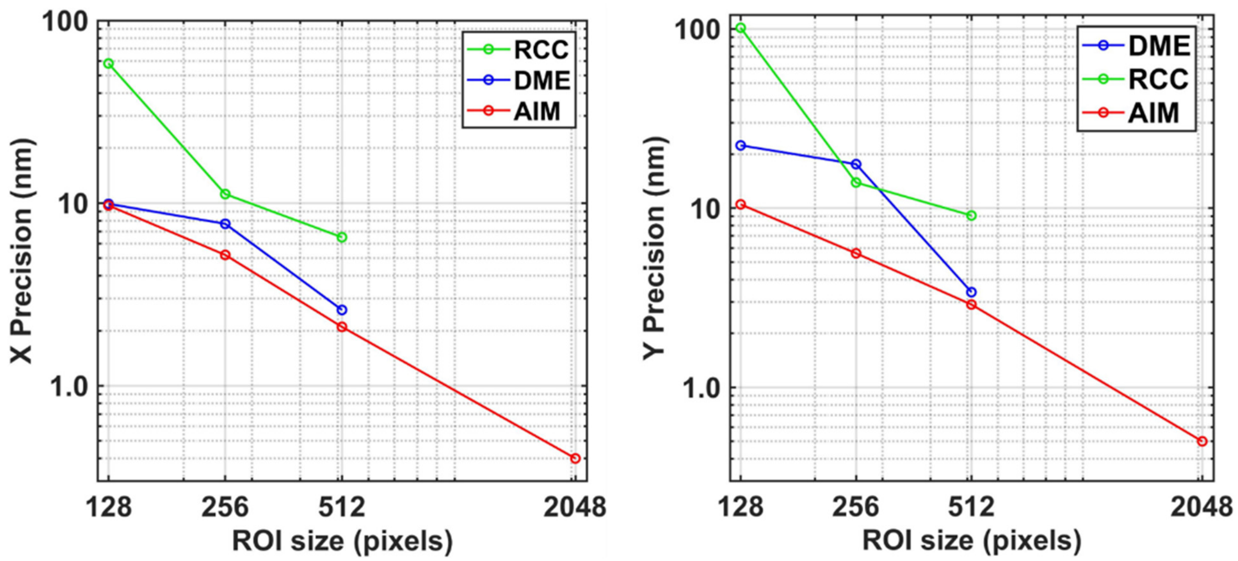

Fig. S4. Drift tracking precision of different algorithms under different ROI sizes for the experimental dataset used in Figure 3. Tracking intervals of AIM, DME, and RCC were 0.2s (20 frames), 0.02s (2 frames), and 0.5s (50 frames), respectively. Considering the long computation time of RCC and DME, we only tested their performance under an ROI size of up to 512×512 pixels. The ground truth drift trajectory is generated by tracking the joint position of ~20 fiducial markers (nanodiamonds) with a tracking frequency of 20Hz. All three algorithms exhibit enhanced tracking precision as the ROI size expands. It is feasible to achieve acceptable precision by carefully selecting ROI size and tuning parameters for a large dataset.

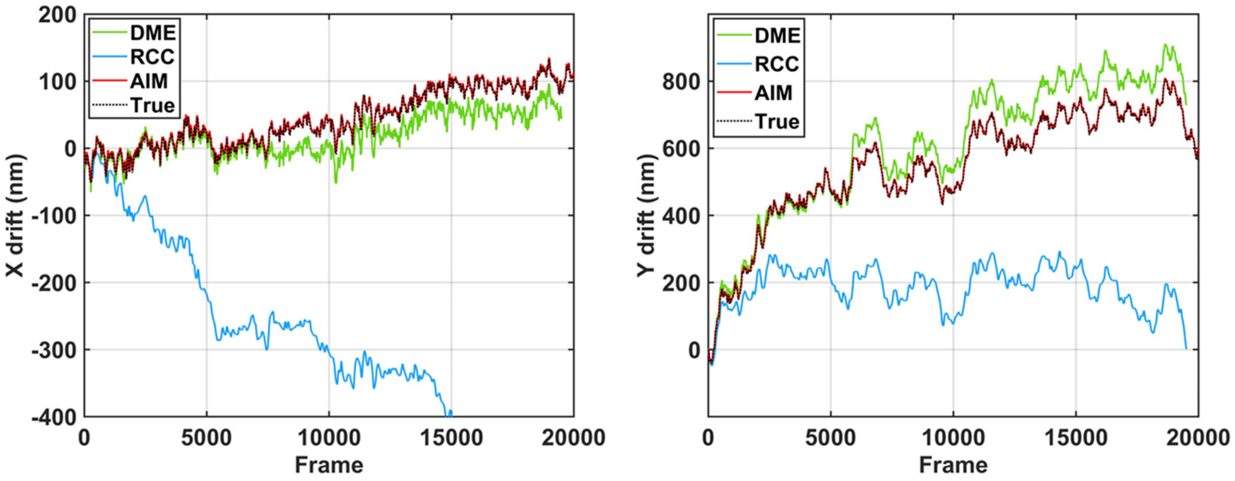

Fig. S5. **Estimated X and Y drift trajectories from the "temporal partition and connect" approach for DME and RCC, in comparison to AIM that uses the full dataset.** The precision for AIM, DME, and RCC is 0.4 nm, 14.2 nm, and 171 nm in the x dimension, and 0.5 nm, 41.7 nm, and 157 nm in the y dimension. Given that the ground-truth drift trajectories are not visually distinguishable from those of AIM. The computational time for AIM, DME, and RCC is 140 seconds, 12 hours, and 4 hours, respectively.

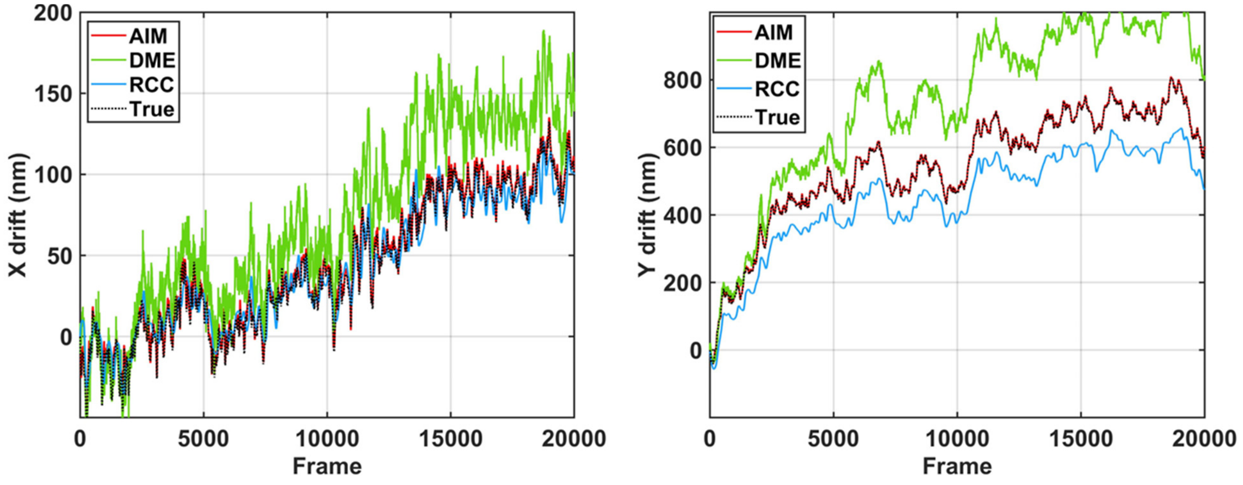

Fig. S6. Estimated X and Y drift trajectories from the "spatial partition and average" approach for DME and RCC, in comparison to AIM that uses the full dataset. The precision for AIM, DME, and RCC is 0.4 nm, 17.9 nm, and 11.3 nm in the x dimension, and 0.5 nm, 62.8 nm, and 23.5 nm in the y dimension. The computational time for AIM, DME, and RCC is 140 seconds, 33 hours, and 27 hours, respectively.

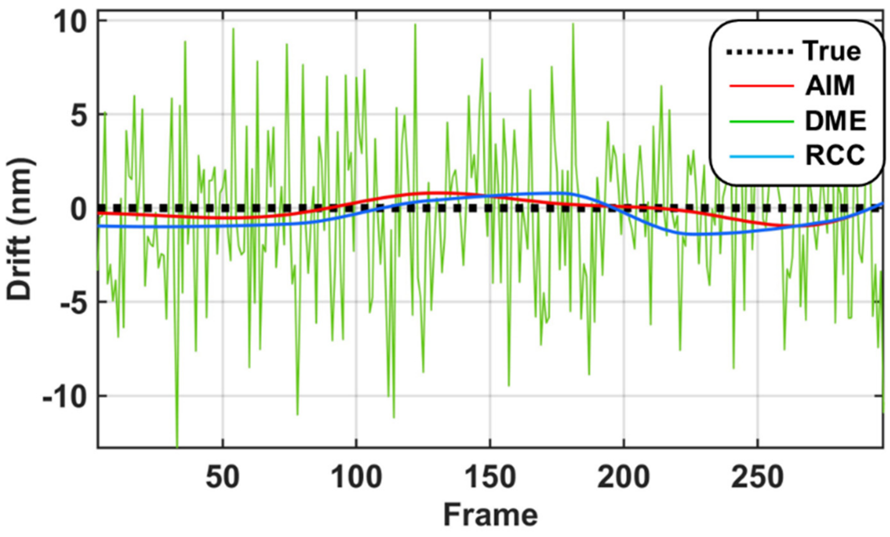

Fig. S7. Drift tracking performance of AIM, DME, and RCC using datasets with minimal drift (with a root mean square drift of 10^{-4} nm/s). Representative drift trajectories for the same dataset were estimated by AIM, DME, and RCC under a localization density of 15 localizations/frame, spatial resolution of 40nm, and zero drift. The tracking interval for AIM, DME, and RCC is 50 frames, 2 frames, and 50 frames, respectively. In nearly drift-free scenarios, DME shows a significantly worse drift tracking precision (5.1 nm) than RCC (1.9 nm) and AIM (1.5nm).

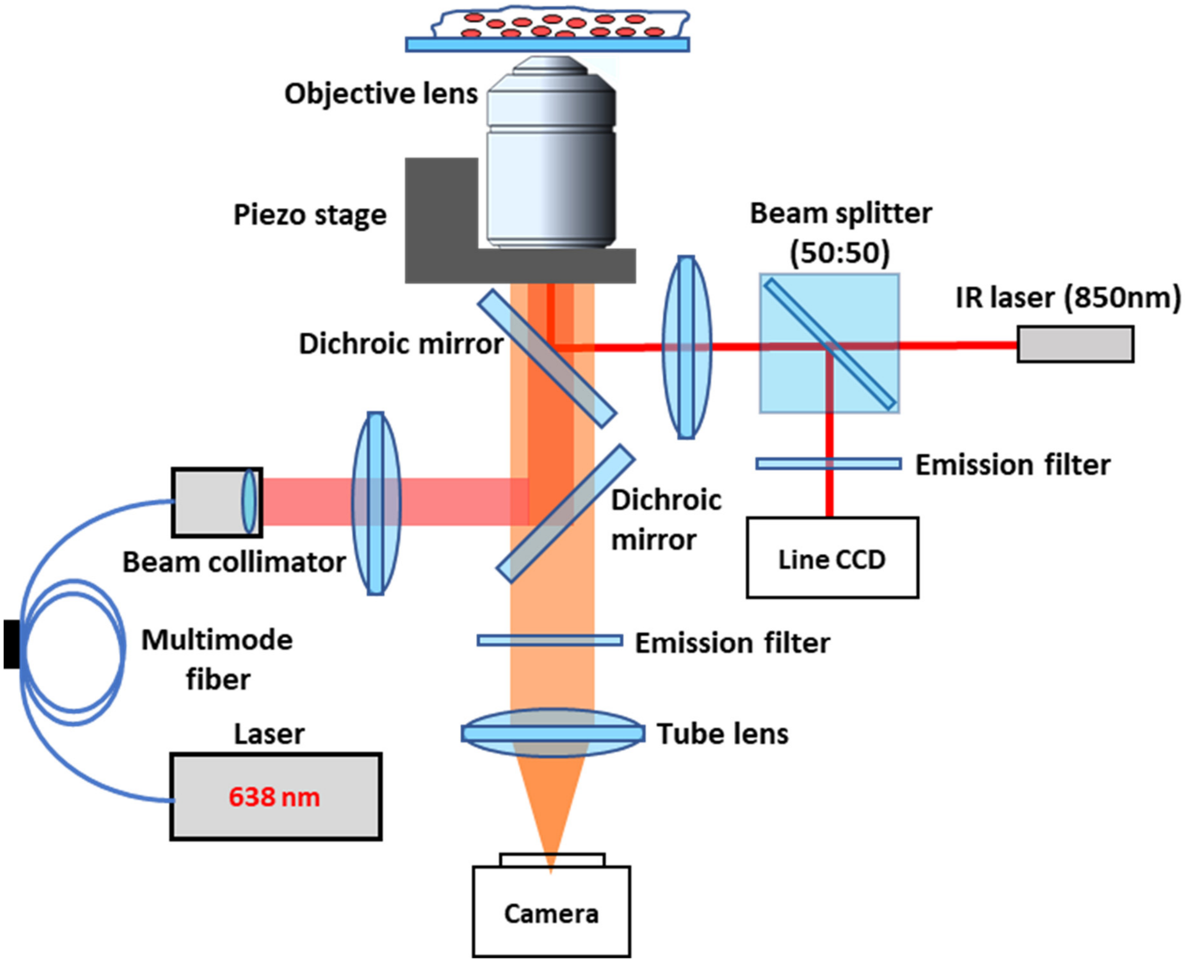

Fig. S8. Schematic of the high-throughput single-molecule localization imaging system used in this study.
